# Macroplastic surface characteristics change during wind abrasion

**DOI:** 10.1038/s41598-025-02738-w

**Published:** 2025-05-21

**Authors:** Isabelle Finlay, Joanna E. Bullard, Lucrecia Alvarez-Barrantes, Sam Davis

**Affiliations:** 1 Geography and Environment, Loughborough University, Loughborough, Leicestershire LE11 3TU UK; 2 Loughborough Materials Characterisation Centre, Department of Materials, Loughborough University, Loughborough, LE11 3TU UK

**Keywords:** Biogeochemistry, Environmental sciences

## Abstract

Mechanical abrasion is an important wind driven process which can degrade plastic litter on sandy beaches, desert environments and in agricultural settings. Wind-driven particle impacts can cause surface roughening and chemical changes and eventually complete fragmentation in high stress environments. Aeolian abrasion has been considered in the context of microplastics (< 5 mm) which can be easily mobilised by wind. However, macroplastic (> 5 mm) abrasion has primarily been confined to engineering studies using high air velocities (> 25 m s^−1^) and large abraders (> 6 mm) which generate greater impact forces than observed in the natural environment. Using laboratory abrasion experiments, we demonstrate that the surface microtextures and surface chemistry of three different types of plastic are substantially altered during the processes of aeolian abrasion at impact particle velocities of 0.6 m s^−1^. After ten days of continuous abrasion with four different erodents the macroplastic surfaces developed textures resulting from micro-cutting, denting, flaking, micro-pitting and surface flattening. The prevalence of each surface texture was dependent upon the angularity of the erodent and the type of plastic. In all cases, polymer surface chemical compositions became more complex due to embedding of shattered abrasive and the replacment of carbon with oxygen and silica.

**Supplementary Information:**

The online version contains supplementary material available at 10.1038/s41598-025-02738-w.

## Introduction

Global production of plastics has increased exponentially since 1950 and alongside this has been an increase in concerns about plastic mismanagement, littering and associated environmental impacts^1,2^. Plastics are very resistant to complete degradation and can have a long residence time in the environment due to their physical and chemical stability^3^. There is no agreed duration for the time required for complete degradation, but it is estimated to range from hundreds to thousands of years^4^. Despite this overall resistance, the initial breakdown of macroplastics (> 5 cm) to smaller components can take place rapidly through physical fragmentation and mechanical abrasion^5^. Physical fragmentation occurs when macroplastics are broken into smaller particles through mechanical (e.g. wind, wave and water action) and thermal (e.g. heating and cooling by solar radiation or fire) processes, and can occur alongside mechanical abrasion which results in the removal or alteration of surface material by external impacts^6,7^. The surface chemistry of plastic may also be changed by photodegradation, the removal of chemical additives or embedding of abrasive particles, any of which can, in turn, alter the effectiveness of mechanical wear^8,9^.

The focus of this paper is the role of the wind in macroplastic breakdown through mechanical abrasion. Plastic debris is common in environments where there is a strong likelihood of exposure to wind erosion and aeolian abrasion, one of the main environmental agents of which is sand. Beach and coastal sand dune systems can accumulate stranded plastics through poor waste management within river catchments^10^ and on shorelines or by littering from recreational activities^11,12^. Shoreline plastic also originates from ocean-based pathways, for example macroplastic litter from fishing activities can be washed onshore after storms and accumulate in strandlines^13,14^. A less well understood, but also important, pathway is the role of the wind in transporting both macro- and micro- plastics potentially very long distances including into remote areas such as deserts and polar regions^15–18^. During near-surface wind transport, plastics may undergo abrasion through intermittent or sustained contact with different natural or anthropogenic surfaces and sediments^9,19^. More extensively, plastic mulch films are used in agriculture and although they are typically removed after harvesting, they can break down in situ and complete removal is difficult leaving plastic debris in soils where it can be redistributed or further broken down during wind erosion^8,20^. It is not necessary for plastics to be fully mobile in the wind to undergo abrasion. Partially buried or entangled macroplastics are common in soils or on beaches and the subaerial component will be exposed to flutter and axial stresses, mechanical abrasion and photodegradation^8,21,22^, whilst the buried portion is protected from these processes.

Near-surface wind-transported sediments move in ballistic trajectories, initiating the movement of a layer of particles close to the surface via creep or saltation; particles travelling higher in the atmosphere move in suspension. Microplastics (< 5 mm) can be preferentially eroded by wind from soil and sand surfaces^17,20^ and exhibit all modes of aeolian transport due to their low density and small surface area^23^. Repeated entrainment, deposition and collisions with sediments suspended in the airflow has been observed to alter the surface of microbeads, microfibres and pellets by abrasion^9,24–26^. Macroplastics are not mobilised by the wind in the same way as microplastics due to their larger size and complexities introduced by material and shape^18^, however surface abrasion will occur from plastic travelling by intermittent saltation over rocks and sediments, or from wind-driven grains impacting the surface of both mobile and stationary macroplastics^24^.

Most current understanding of macroplastic abrasion originates from polymer engineering research to improve the performance and functional life of materials^27^. Studies using dry rubber sand wheels and sandblasting by abrasive particles have observed a range of abrasive marks on the surface of polymers, which are generally accepted to be a function of material strength and abrader impact angle^28,29^. Polymers of a ductile nature typically wear by micro-cutting, while brittle polymers form cracks and pits on the surface^30,31^. The extent of wear is a function of erodent size, until a critical limit is reached and the mass per grain reduces the abrasive particles’ lift duration and applied load^32^. Abrader angularity is also important, with rounded or spherical materials causing less abrasive wear than angular particles due to the lack of ‘edges’ and fewer impact points^25,33^. As well as surface change, cumulative impacts can result in the fragmentation of macroplastic into secondary micro- and nano- plastics. Brittle polymers show a linear erosion rate, with microplastics produced immediately after the onset of abrasion, while ductile polymers exhibit an incubation period with minimal mass loss as energy is expended in roughening the surface^32,34,35^. Embedding of the abrasive particle can also reduce fragmentation rates by shielding the target material from impacts^36^.

Threshold wind speeds required to entrain sand-sized mineral particles in the natural environment range from 4 to 8 m s^−1^, which is substantially lower than the velocities used in tribological studies (25–60 m s^−1^)^35,37,38^. Although natural wind velocities of this magnitude have been recorded, they are rarely sustained for extended periods. Furthermore, the sliding speeds used by sandpaper abrasion tests (200−800 rpm) generate larger frictional impacts than observed naturally^39,40^. Macroplastic samples are also often fully fixed down and unable to repel against abrading particles as occurs in the natural environment. The fragmentation of macroplastic (high-density polyethylene) into microplastics under UV exposure has been emulated in a controlled sandy beach environment^21^, however, macroplastic breakdown by wind-driven mechanical abrasion in dry, sandy environments is yet to be fully explored. Research in this field will provide insights into the development of surface texture and wear patterns on macroplastic surfaces, and the generation of fine plastic fragments over time^41^. Determining the size of environmental plastics is integral to discerning their impacts, as macroplastics can cause entanglement, drainage system blockage and loss of aesthetics, while microplastics can be inhaled and damage internal organs^42,43^. Surface texture is important because roughened and porous plastic surfaces can absorb heavy metals which can have toxic effects if ingested^43^.

To examine the effects of wind-driven sand abrasion on microplastic surfaces under natural threshold wind conditions, low-density polyethylene (LDPE), polyethylene terephthalate (PET) and expanded polystyrene (EPS) were placed in an aeolian abrasion simulation chamber for ten days (see full Methods for details). Each type of plastic was abraded with three types of abrasion media of different sizes and angularities comprising well-rounded glass beads (GB) and rounded (SR_149_), angular (SA_256_) and very angular quartz particles (VA_357_). The physical surface micro-textures, abrasion wear signatures and surface chemistry of the macroplastics before and after abrasion were characterised using a high-resolution Scanning Electron Microscope (SEM) and Energy-dispersive Spectroscopy (EDS).

## Results

### Physical surface change

**Fig. 1:**
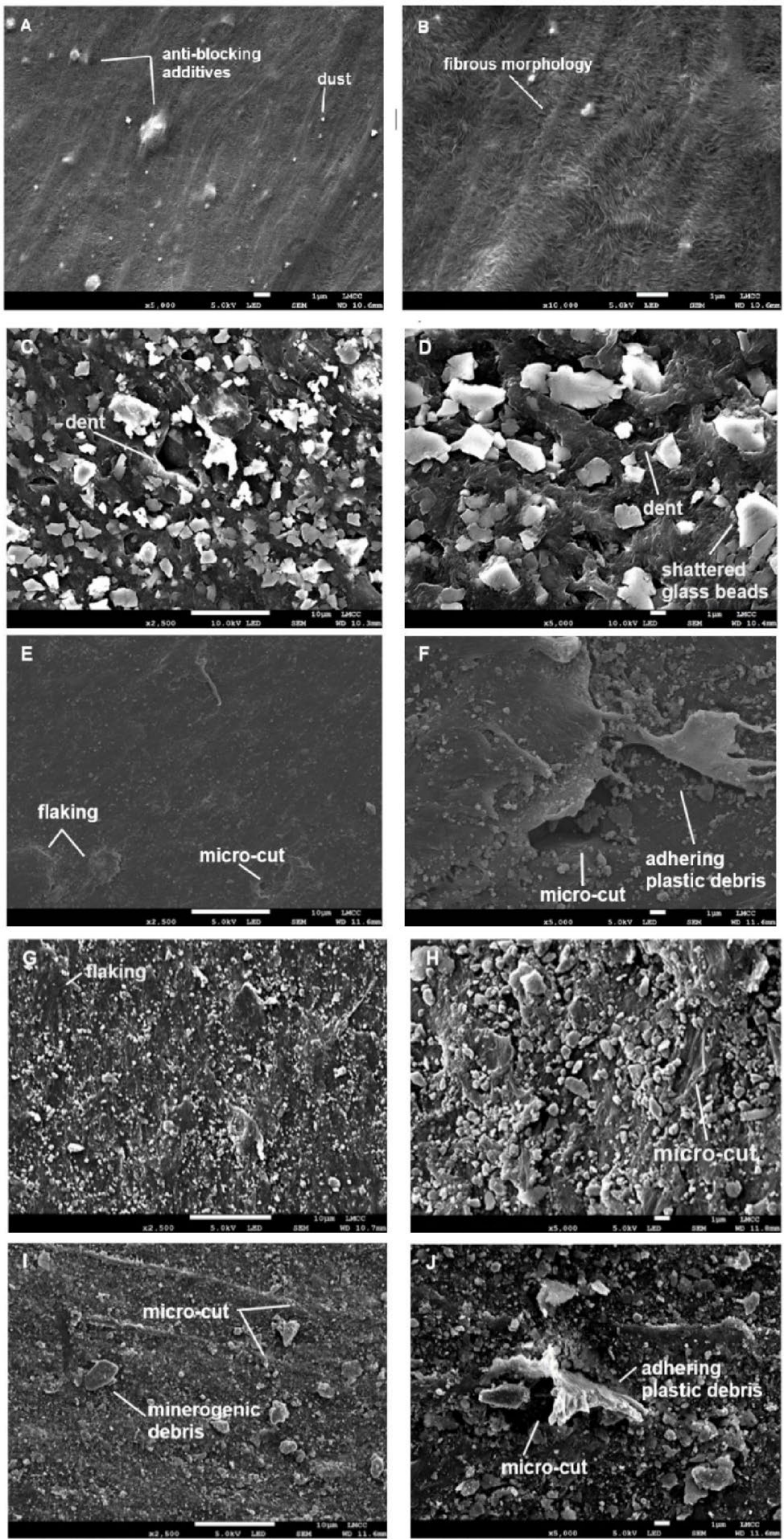
Scanning electron micrographs of unabraded LDPE (**A**, **B**), and LDPE following 10 hours of abrasion with glass beads (**C**,**D**), rounded desert sand (**E**,**F**), beach sand (**G**,**H**), angular commercial sand (**I**,**J**). For A,B white scale bar represents 1 µm. For all other samples, in images on the left the white scale bar represents 10 µm and in images on the right the white scale bar represents 1 µm.

Ten days of aeolian abrasion was sufficient to cause visible, textural changes to the surface characteristics of all three types of plastic regardless of the abrasive used. Prior to abrasion, LDPE exhibited a smooth surface texture with a branched polymer structure characteristic of a semi-crystalline plastic (Fig. 1). Contamination of the surface by dust particles and anti-blocking additives, used to impart a microporous texture to reduce adhesion between film layers, was also visible on the unabraded surface. Abrading the LDPE surface with glass beads caused 51.03% of the surface to wear and the formation of shallow dents on the polymer surface with material displaced in-front of the impact in a squeezed lip morphology.

Abrading LDPE with sand of increasing angularity caused a change in abrasion signatures from denting to micro-cutting (Fig. 1). SR_149_ caused abrasion of ≈ 24.39% of the LDPE surface. Early stages of deformation were evident from a large degree of matrix flaking. In areas of higher stress, micro-cuts developed with a mean length of 4.06 ± 4.14 μm. Loosely adhering plastic debris from the cut areas appeared as curled flakes on the surface, in some cases these had become detached allowing the deformed area to fill with mineral debris detectable from their chemistry using EDS. SA_256_ caused more extensive surface abrasion affecting ≈ 60.9% of the area examined, particularly through surface flaking and the formation of larger cuts with a mean length of 12.98 ± 5.73 μm. VA_357_ sand abraded ≈ 38.58% of the LDPE surface causing longer micro-cuts than the less angular sediment ($\bar{x}$ = 15.65 ± 12.61 μm). The adhering plastic debris from cut areas was observed to be highly curled and crumpled from successive impacts. On all abraded surfaces embedded minerogenic debris, created during wear of the abrasive material from plastic-sand and sand-sand collisions during the experiment, was present.

**Fig. 2:**
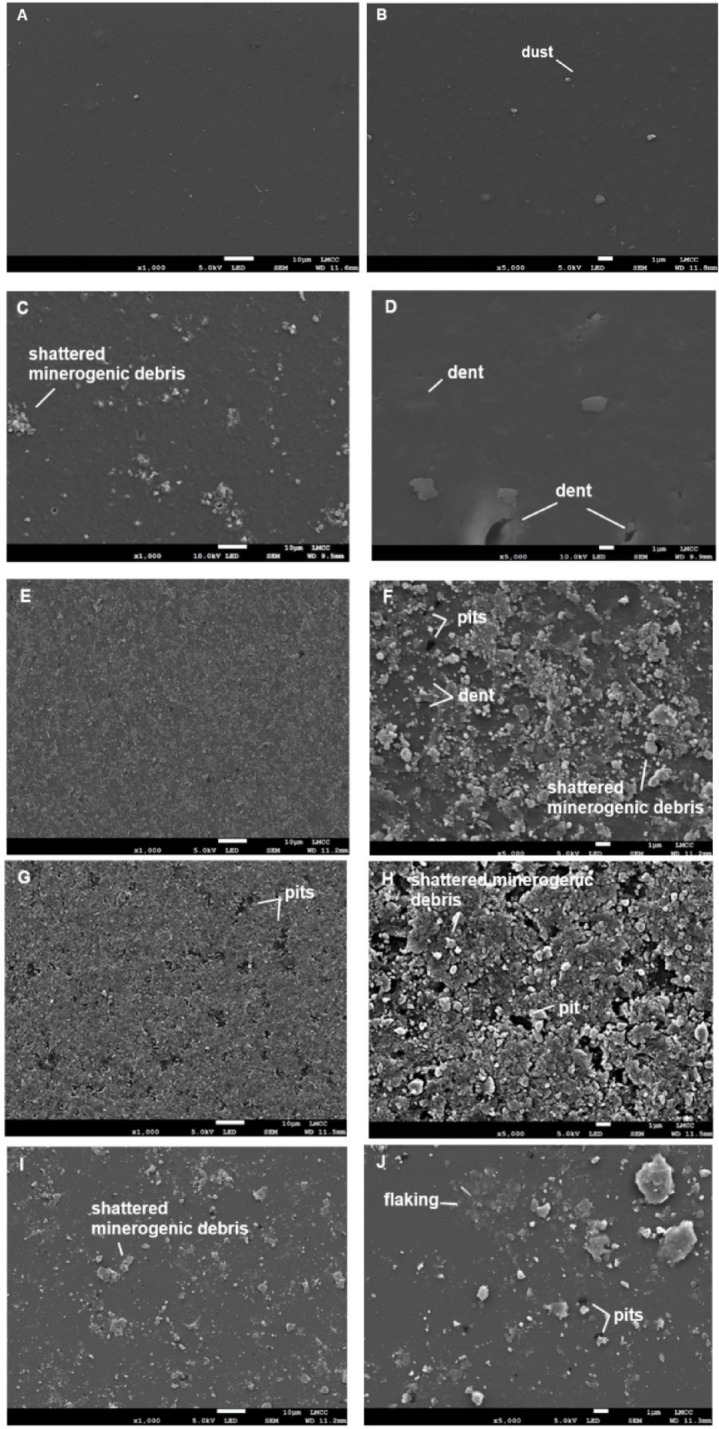
Scanning electron micrographs of unabraded PET (**A**, **B**), and PET following 10 hours of abrasion with glass beads (**C**,**D**), rounded desert sand (**E**,**F**), beach sand (**G**,**H**), angular commercial sand (**I**,**J**). For A white scale bar represents 10 µm; for B white scale represents 1 µm. For all other samples, in images on the left the white scale bar represents 100 µm and in images on the right the white scale bar represents 10 µm.

Unabraded PET exhibited a smooth surface with fine minerogenic particles covering 0.7% of the surface area (Fig. 2). The glass beads caused denting on ≈ 11.91% of the PET surface. Dents were smooth and shallow with a spherical morphology and mean diameter of 1.75 ± 0.62 μm. Thin fragments of plastic debris were also scattered across the macroplastic surface. SR_149_ sand caused abrasion of ≈ 41.85% of the PET surface which also appeared highly dented. Dents were a similar size to those created by glass beads ($\bar{x}$ diameter 1.66 ± 0.95 μm) and were smooth with material displaced to the side of the compressed impact zone in a shear lip. Smaller debris-free erosion pits ($\bar{x}$ = 0.54 ± 0.21 μm) formed in some areas. Flaked plastic debris was also present across the unpitted macroplastic surface. PET abraded with SA_256_ developed erosion pits ($\bar{x}$ = 1.55 ± 1.5 μm) as the dominant wear texture, and these covered ≈ 37.25% of the surface. The pits were mostly free of minerogenic debris, however between the erosion pits the surface matrix was shattered, with fragments of plastic debris and embedded minerogenic particles. VA_357_ sand created less surface change than the other two sands causing detectable abrasion of only ≈ 13.27% of the PET surface. Small erosion pits were present ($\bar{x}$ diameter 0.96 ± 0.54 μm) with some abrasive minerogenic debris lodged inside. Early stages of polymer matrix flaking was also evident in less impacted areas, creating a thin deformed layer of plastic on the surface.

**Fig. 3:**
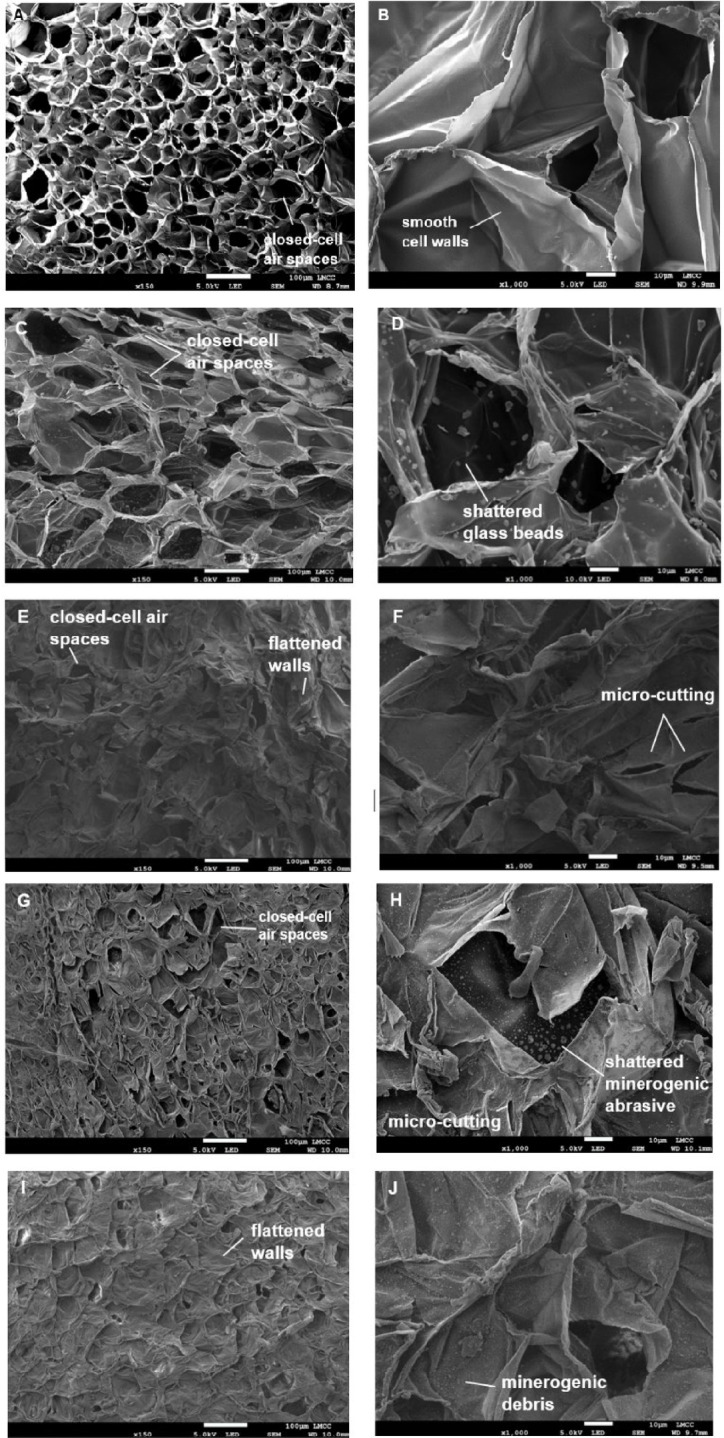
Scanning electron micrographs of unabraded EPS (**A**,**B**), and EPS following 10 h of abrasion with glass beads (**C**,**D**), rounded desert sand (**E**,**F**), beach sand (**G**,**H**), angular commercial sand (**I**,**J**). For images on the left the white scale bar represents 100 μm and in images on the right the white scale bar represents 10 μm.

Unabraded EPS comprised a structure of closed cell air spaces with thin adjoining walls ranging in diameter from 18.71 μm to 103.21 μm ($\bar{x}$ = 51.42 μm). The air spaces made up ≈ 38.83% of the surface layer and internal base of the cell had a smooth texture. Abrasion with glass beads flattened the cell walls and reduced the number of open air cells to 29.32% with a concomitant narrowing of the mean diameter of the remaining cells (43.85 ± 18.87 μm). The inner walls retained a mostly smooth texture, however the outer surface layer appeared crumpled. Shattered minerogenic debris also covered the plastic surface and penetrated through to the exposed underlying layer (Fig. 3).

Quartz sand caused more flattening of the EPS surface than the rounded glass beads. Abrasion with SR_149_ caused a reduction in the closed-cell ring structure to ≈ 9.85% and resulted in an overall smoother plastic surface due to flattening of the cell walls by impacts. Where EPS cells were still partially intact, their mean diameter was reduced to 28.33 ± 11.59 μm. The outer cell walls were highly curled and crumpled with evidence of micro-cutting on the surface. Following abrasion with SA_256_ sand, ≈ 15.87% of the cells were intact with an mean ring diameter of 35.53 ± 18.67 μm. The outer layers of the walls became rough and crumpled, with evidence of micro-cutting on flattened surfaces. Fine minerogenic particles ($\:\le\:$1 µm) from the worn abrasive adhered to the EPS surface and created impact shatter marks inside the cells. Only 5.4% of closed cell air spaces remained after abrasion with VA_357_ sand. Remaining cells had a mean diameter of 33.17 ± 11.64 μm with a highly wrinkled and torn pattern resulting from compression of the outer walls. Fine particles from shattering of the abrasive also covered the surface.

### Surface chemistry changes

**Fig. 4:**
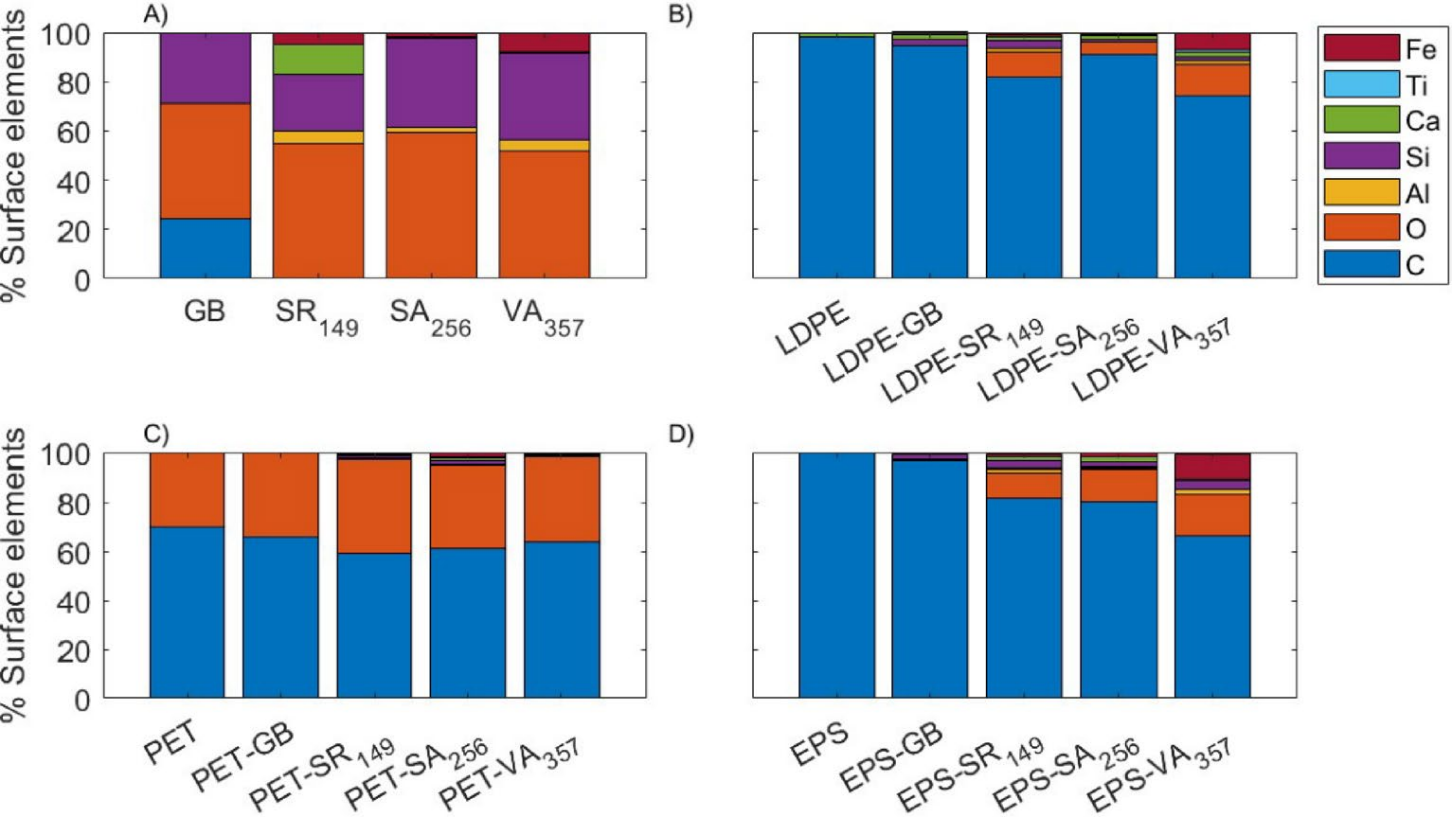
Key surface elements present on (**A**) abrasives used, and unabraded and abraded plastics. (**B**) LDPE, (**C**) PET and (**D**) EPS. For abbreviations see Table 1.

EDS scans of the abrasion media surfaces indicate the glass beads predominantly comprise oxygen (42.23%), silica (25.72%), carbon (21.99%) and sodium (8.14%)(Fig. 4). The three sands used comprised primarily oxygen (50.97 to 59.13%) and silica (21.73 to 35.94%). SR_149_ had a higher % of calcium (11.53%) than the other sediments, and VA_357_ had a relatively high % of iron (7.75%). The surfaces of all four abrasives had small quantities of other elements such as magnesium, potassium, titanium as detailed in Supplementary Information Table S1. In contrast, prior to abrasion, the dominant surface element for all three macroplastics was carbon with LDPE and EPS both > 98% and PET ≈ 70% (Supplementary Information Table S2–4). The other substantial element on the PET surface was oxygen (29.61%).

Ten days of abrasion with different media caused changes to the surface chemistry of the macroplastics but the effects varied for different plastic-sediment combinations (Fig. 4). For all plastics, abrasion with glass beads caused the least amount of change with a reduction in % surface carbon of between 2.8% (EPS) and 4.2% (PET) and the addition of small quantities of silica (< 3%) and other elements (< 0.5% each). For LDPE and EPS there were substantial changes to the plastic surface chemistry following abrasion with sand, with the greatest change resulting from abrasion with VA_157_, the largest and most angular sediment. For both these plastics, the % carbon on the surface was reduced (by 24% for LDPE and 33.14% for EPS) and replaced by oxygen (12.6 to 16.9%), silica (1.66 to 3.55%) and small quantities of other elements that were present on the abrasive but not on the unabraded plastic (e.g. potassium, iron, magnesium). The surface chemistry of the PET sample underwent less change with a 5–10% decrease in carbon, only a slight increase in the % oxygen (3–8%) and minor increases in other elements (typically < 1% change).

There is no statistically significant relationship between the % area of the plastic with visible signs of abrasion and the change in carbon or oxygen content for LDPE (across all tested abrasive media). In contrast, there is a statistically significant negative relationship (*p* < 0.05) between % surface abrasion and % surface carbon for both PET (R^2^ = 0.899 and EPS (R^2^ = 0.888), and a statistically significant positive relationship (*p* < 0.05) between % surface abrasion and % surface oxygen for EPS (R^2^ = 0.870). The size of wear features visible on the microplastic surfaces indicates any secondary microplastics generated during abrasion were < 16 μm for LDPE, < 1.6 μm for PET and < 34 μm for EPS. Plastic debris adhering to the microplastic surfaces was < 5 μm and more typically < 1 μm (Fig. 1).

## Discussion

**Fig. 5:**
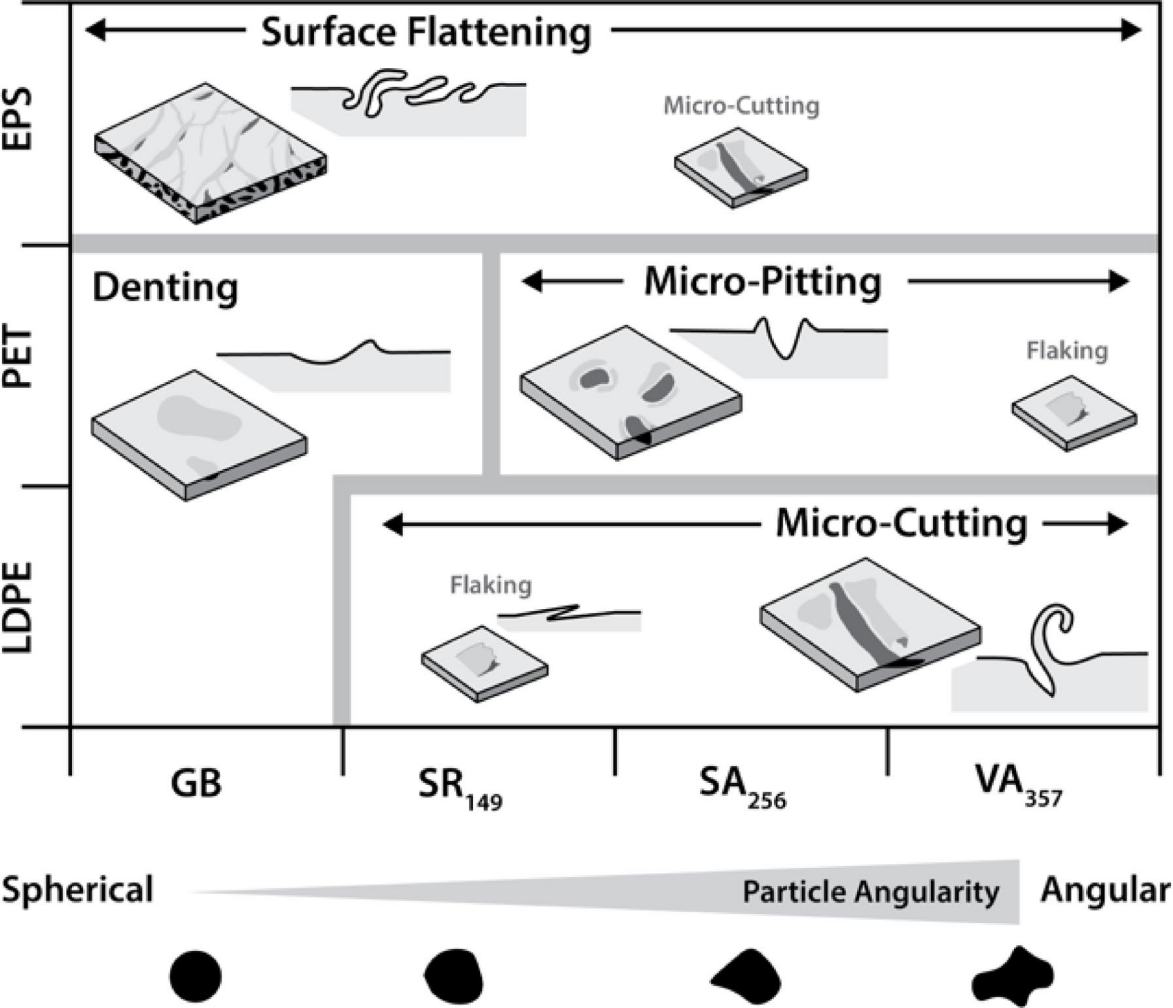
Dominant surface textures observed on each plastic type, following abrasion, as a function of sediment type. In each phase, the major process is indicated in bold. Grey bands represent broad areas of textural transition.

Abrasion resistance is not an intrinsic material property and is highly dependent on the polymer type and abrasive materials tested. The three plastic types used in the present experiments differed in terms of their ductility and crystallinity, and were worn in different ways by aeolian abrasion (summarised in Fig. 5). LDPE exhibited typical micro-abrasive features of a ductile polymer, with micro-cutting as the dominant sign of wear^5^. Abrasion debris largely appeared as adhered plastic flakes on the surface, which is typical of the micro-cutting process with material displaced upward from the zone of impact^7^. PET is also a ductile polymer, but surface morphology change was characterised by dent and pit formation due to the compression and removal of material on impact. Most features appeared empty of minerogenic debris as the relax in strain between impacts caused abrasive particles to shed and either be recirculated within the chamber, or removed through the air outlet. PET is a harder polymer than LDPE with greater efficiency to repel impacting particles. The depth of indentation marks is likely more important than the lengthwise deformation for harder polymers, as a greater force is required for abrasive particles to drag across the surface^44^. Abrasive signatures on both PET and LDPE typically developed as discrete features owing to the semi-crystalline nature of ductile polymers which have uneven tensile strength across the surface. Stress is concentrated in crystallised areas which likely explains why deformation features were isolated and not coalescing.

For ductile polymers, particle shape influences the pattern of surface deformation^33^. Spherical glass beads with minimal surface micro-texture caused denting of LDPE and PET surfaces, displacing material to the side or in front of the impact. Denting from round material has been observed in several tribological studies, with spherical steel balls exhibiting less cutting action than angular sand grits due to the lack of edges and contact points^45^. Quartz sand grains caused micro-cutting and micro-pitting by removing material due to the sharp edges^46^. For LDPE, VA_357_ created longer micro-cuts ($\bar{x}=$15.69 μm) than SA_256_ ($\bar{x}=$12.98 μm) and SR_149_ sand ($\bar{x}=$4.06 μm) due to the larger number of focused contact points on the sediments, creating more drag than blunter surfaces^47^. Angular grains also deposited more minerogenic debris and dust on the polymer surface due to the progressive chipping and spalling of edges during abrasion^48^.

The size of the impacting particle also influences abrasive wear. For LDPE, greatest surface roughening was caused by SR_149_ (60.9%) and not VA_357_ (38.58%) despite its larger size. For a constant airflow, the effectiveness of abrasive wear increases with particle size until a level of saturation is reached where the increasing mass per grain reduces the sediment’s lifting duration and velocity^49^. Consistent with this, for a constant air inflow velocity, the saltation cloud height for VA_357_ (3 cm) was lower than that of SA_256_ (6 cm) and SR_149_ (8 cm) with sand particles with higher D50 and greater mass travelling at lower particle velocities. Additionally, fewer grains are needed to reach the unit weight of a sample with larger particle sizes which reduces the probability of the abrasive colliding with the plastic. For example, in these experiments, the chamber contained approximately 22 million particles of SR_149_ (in 100 g) compared with only 1.59 million particles of VA_357_. It is likely that SA_256_ also caused more abrasion and formed larger pits (1.66 μm) on PET compared to VA_357_ (0.96 μm) as particle velocity and the number of impacts were optimal to cause permanent metamorphic damage^25^.

The characteristic of EPS mean that it can be highly ductile (under compression) but becomes brittle under tension. The initial surface structure of the EPS tested here was very different to that of the LDPE and PET, being characterised by closed cell air spaces that became flattened during abrasion due to the compression of the thin cell walls. Flattening was largely homogenous across the surface, likely due to the amorphous nature of EPS with even stress distribution^32^. Abrader angularity exhibited little influence on deformation characteristics with flattening dominant for both glass beads and angular sands. However, the extent of surface abrasion did increase with angularity and the more angular sand also caused micro-cutting from ripping of the cell outer layer^6^. EPS abraded with SA_256_ had the largest closed cell air space diameters indicating less abrasion had occurred compared to with other media, however, it is likely the optimal velocity and number of impacts due to the size of SA_256_ created a transition from surface flattening to peeling and separation of the flattened surface layer^50^. This would explain the larger diameter of open spaces and shattering of abrasive identified below the surface on the underlying layer as the surface is peeled back.

The surface microtextures observed here – pits, micro-cracks, dents, flakes and cuts - are indicative of mechanical weathering and have also been observed to occur following macroplastic exposure in aqueous environments including lakes, saltmarshes and coastal zones^51–53^. As with our simulated aeolian environment, studies of surface texture development on plastics in aqueous environments have found differences between types of plastic, for example polypropylene and polystyrene develop abrasion features more rapidly than high density polyethylene^53^. In the initial stages of polymer abrasion, an incubation period may occur in which little or no mass is lost as impact energy is dissipated in roughening the target surface^35^. Embedding of fragmented sand particles occurs preferentially during the incubation period, which shields the surface from subsequent impacts^54^. Hence, the abrasive debris on the plastic surfaces may have reduced the effective abrasion rate^36^. The presence of small adhering particles – both minerogenic and plastic – is also widely observed, and in environments where biofilms develop on the plastic surface these are suggested to be associated with biofilm remnants^52^. In our aeolian study no biofilms were developed on the plastics and the adherence of fine particles is thought to be by physical embedding, or possibly tribocharging^55^.

An influential factor on abrasion signatures, not quantified here, is expected to be the angle of impact of the abrader^29^. For example brittle polymers undergo most abrasion when impacted at a 90° angle when the tangible impact force is greatest^35^. In contrast, micro-cutting in ductile polymers peaks at low impact angles between 10° and 30°. The impact angle of the abrader in the experimental chamber used here will vary according to the size of particle used and air inlet velocity, which combined determine the saltation cloud height, as well as the shape, and relative height and angle of the plastic compared to the impacting particles. The variability in rates of surface texture development associated with saltation impact trajectories in a natural aeolian environment remains a subject for further research.

Plastics are largely composed of carbon but are rarely pure and contain a variety of additives to enhance their functionality. Carbon made up > 98% of the surface of LDPE with the remainder comprising small quantities of calcium (1.22%), titanium (0.41%) and aluminium (0.04%) which are used as pigments or additives to increase mechanical strength^56^. The total number of elements detected on the surface following abrasion ranged from 4 (glass beads) to 9 (VA_357_). Cut and deformed layers created by angular sand can be chemically different to the original plastic, and oxygen from the oxygen rich sediments can diffuse through the thin surface layer, reducing the share of carbon in the mass content analysis^57^. In line with other studies using quartz sand as abraders, silica was transferred onto the plastic surface in small concentrations due to the embedding of minerogenic debris produced by the fragmentation of abraders or chipping of rough edges^9,25^. Quantities of other elements were highly variable and showed no obvious relationship to the respective amount of each element on the abrader, with not all elements being transferred in some cases.

PET was composed of carbon (70.17%), aluminium (0.02%) and molybdenum (0.2%) which is often used a corrosion inhibitor. Unlike the other plastics tested, oxygen was already present on the unabraded surface (29.61%). Due to its even distribution, it is possible that oxygen plasma treatment was used as a wettability enhancing agent for the use of the PET as a bottle^58^. The surface carbon content of PET showed a strong decline as the % surface abraded increased. Dents and micro-pits form localised areas of increased surface porosity, increasing the surface area and enabling the binding of new elements to the surface^59^. This might explain the presence of small quantities of elements on the abraded PET which were not present on the unabraded PET.

EPS had the largest surface carbon content (99.56%) with traces of aluminium (0.07%) and molybdenum (0.37%) as stabilisers. As metal additives are used to achieve the final desired plastic properties, the complete removal of molybdenum on all the unabraded surfaces suggests a decline in functionality of the polymer properties following abrasion and risk of fragmentation into inhalable microplastics in the environment^60^. As with the other polymers tested, EPS abraded with glass beads had fewer elements than sand abraded surfaces due to the fewer elements present on the erodent surface. On the original surface and EPS abraded with glass beads, no oxygen was detected which is surprising given the large amount of closed cell air spaces. However, due to EDS only being able to measure elements up to a depth of 5000 nanometres, the elemental composition in the deep closed cell air spaces is likely not accounted for.

Macroplastic abrasion in the present study has presented as deformation features on the polymer surfaces. Based on the size of these signatures, it is anticipated that any fine plastic particles produced in the abrasion process will be in the micro- and nano- plastic size ranges. Nanoparticles can be inhaled and, in sufficient quantities, can have toxicological and pathological effects on human health and that of other species^43^. Continuous particle movement in the chambers for 10 days equates to approximately 5 months of continual saltation in the environment at threshold wind velocity (≈ 6 m s^−1^). Therefore, macroplastic situated even within the maximum density saltation layer in the environment will have a long residence time before being fragmented into microplastics or nanoplastics by mechanical abrasion by sand.

## Methods

### Abrasion chamber

A widely-used test-tube design of abrasion chamber was used for all experiments^61^ (Supplementary Figure S1). A 5 cm x 10 cm sample of the plastic to be abraded was loosely taped to the side of the chamber and 100 g of the abrasive media was added to the bottom of the chamber. This is a much higher quantity of erodent than in other studies using this chamber design (usually ≈ 10 g)^48,61^ and was used to ensure that the lowest portion of the plastic sample was always buried. This allowed the plastics to flex on impact but reduced flutter. Air was fed into the chamber by a pump to lift particles into saltation from the centre and impact the inward-facing surface of the macroplastic. Airflow exited the inlet tube into the chamber at 11.7 m s^−1^ which was sufficient to form a saltation cloud, the height of which varied according to the abrasion media (Table 1). Sediment particle velocities within the chamber, measured using a a Dantec™ 2D Laser Doppler Anemometer, are c. 0.6 m s^−1^ which is similar to near-bed particle velocities for saltating sand in wind tunnel studies^61^. Mechanical power input to the grains is estimated to be 0.05 W kg^−1^ which, for continuous particle movement within the chamber for 10 days, equates to approximately 5 months of continual saltation in the field at threshold velocity (6 m s^−1^)^55^. All air exiting the chamber was fed into a sealed deionized water bath to capture any particles produced and prevent them from contaminating the air circulating through the vacuum pump. The mass of fine particles captured varied by plastic-abrasive combination and varied from 0.003 to 0.069% of the initial sample weight (100 g) (Supplementary Tables S2–S4). It was not possible to separate micro- and nano- plastic particles generated during abrasion and removed through the air outlet from the fine particles generated by wear of the abrasion media.

**Table 1:** Summary of erodent properties used in abrasion experiments. See also supplementary figure S2.

Erodent	Abbreviation used in text	Material	Sediment size (µm)			Roundness^63^	Height of saltation cloud (cm)	Surface texture features^64^
			D10	D50	D90			
Laboratory grade glass spheres	GB	borosilicate	279	382	514	well-rounded	≈ 3	Very low relief microtopography; minor small adhering particles
Desert sand	SR_149_	quartz	89	149	252	rounded – sub-rounded	≈ 8	Low relief microtopography with bulbous edges; minor dissolution features
Beach sand	SA_256_	quartz	178	256	365	sub-rounded - angular	≈ 6	Glacio-aeolian sediments, bulbous edges, mechanically-upturned plates and weathered pits
Commercial sand	VA_357_	quartz	150	357	685	angular – very angular	≈ 3	Sharp microfeatures including triangular-faceted edges, v-shaped cracks

### Macroplastics and erodents

The majority of plastic debris found in sandy beach or desert environments consists of single use plastic packaging made from a range of polymer types^62^. Three types of plastic were selected for the abrasion experiments based on the reported prevalence in shoreline environments^63^ These were a low-density polyethylene supermarket bag (LDPE), a drinks bottle made of polyethylene terephthalate (PET) and a Styrofoam cup made of expanded polystyrene (EPS). The selected materials encompass both brittle and ductile polymers with amorphous and semi-crystalline structures to ensure heterogenous mechanisms of degradation. Each plastic was abraded with four different erodents of various sizes and angularity. These were chosen to represent a range of different natural environments. The erodents comprised well-rounded, spherical borosilicate glass beads (GB), rounded, quartz desert sand (SR_149_), a glacio-aeolian beach sand (SA_256_) and a commercial sharp sand (VA_357_) – where the subscript indicates median particle size (D50). The properties of the erodents are summarised in Table 1 and Supplementary Figure S2.

### Surface characterisation

 At the end of the abrasion period, each macroplastic sample was rinsed gently with deionised water to remove any large plastic or sediment particles to allow an examination of the surface properties and structure. A sample of each abraded plastic extracted using a scalpel from the area of maximum abrasion as well as an unabraded sample were examined using a Scanning Electron Microscopy (JSM-7800 F analytical field emission scanning electron microscope - SEM) with 5 kV electron accelerating voltage. All samples were coated with gold and palladium before analysis to reduce surface charging. Energy-dispersive X-ray spectroscopy (EDS) was used to map the surface elemental composition of the abraded and unabraded plastics as well as each of the abrasive materials. SEM images were analysed using Image J (1.38 e/Java_1.5.0_09). A series of measurable metrics were selected for each plastic to quantify abrasion, including micro-cutting length and pit and dent diameter^60^. The % of surface abrasion was approximated by adjusting the image threshold of a ×2500 magnification image so that abraded features and adhering particles had a high contrast. The ‘analyse particles’ function was used to calculate the area of high contrast features, expressed as a % of the total image area. As unabraded expanded polystyrene already exhibited an uneven surface with open air spaces, the extent of surface abrasion was quantified by the amount of surface flattening and air space reduction. The size of micro- and nano-plastic particles generated by abrasion was estimated from the size of wear scars on each macroplastic sample and the size of plastic debris visible on the micrographs.

## Electronic supplementary material

Below is the link to the electronic supplementary material.

Supplementary Material 1

Supplementary Material 2

Supplementary Material 3

## Data Availability

The data collected during this study are available in the article and supplementary material.
