# Supplementary material for: Macroplastic surface characteristics change during wind abrasion
Source: Sci Rep. 2025 May 21;15:17630. doi: 10.1038/s41598-025-02738-w (PMC12095662; doi:10.1038/s41598-025-02738-w)
Supplement: Supplementary file 2 — Supplementary Material 2 [file 41598_2025_2738_MOESM2_ESM.pdf]

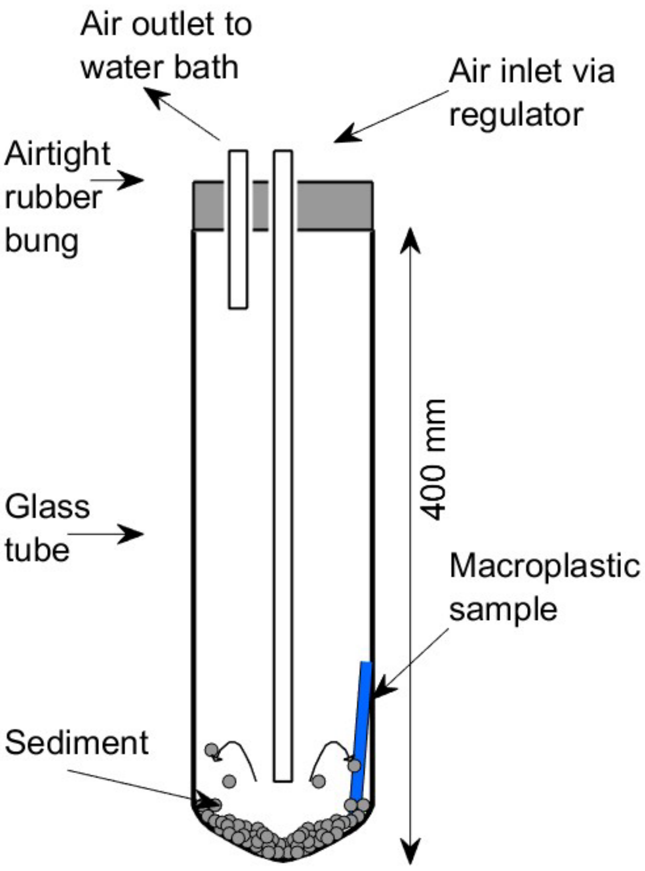

Supplementary Figure S1: Schematic diagram of abrasion chamber apparatus used for the experiments. Air velocity at the base of the inlet is 11.7 m s-1. Sediment particle velocity within the chamber is c. 0.63 m s^{-1}
