# Supplementary material for: Macroplastic surface characteristics change during wind abrasion
Source: Sci Rep. 2025 May 21;15:17630. doi: 10.1038/s41598-025-02738-w (PMC12095662; doi:10.1038/s41598-025-02738-w)
Supplement: Supplementary file 3 — Supplementary Material 3 [file 41598_2025_2738_MOESM3_ESM.pdf]

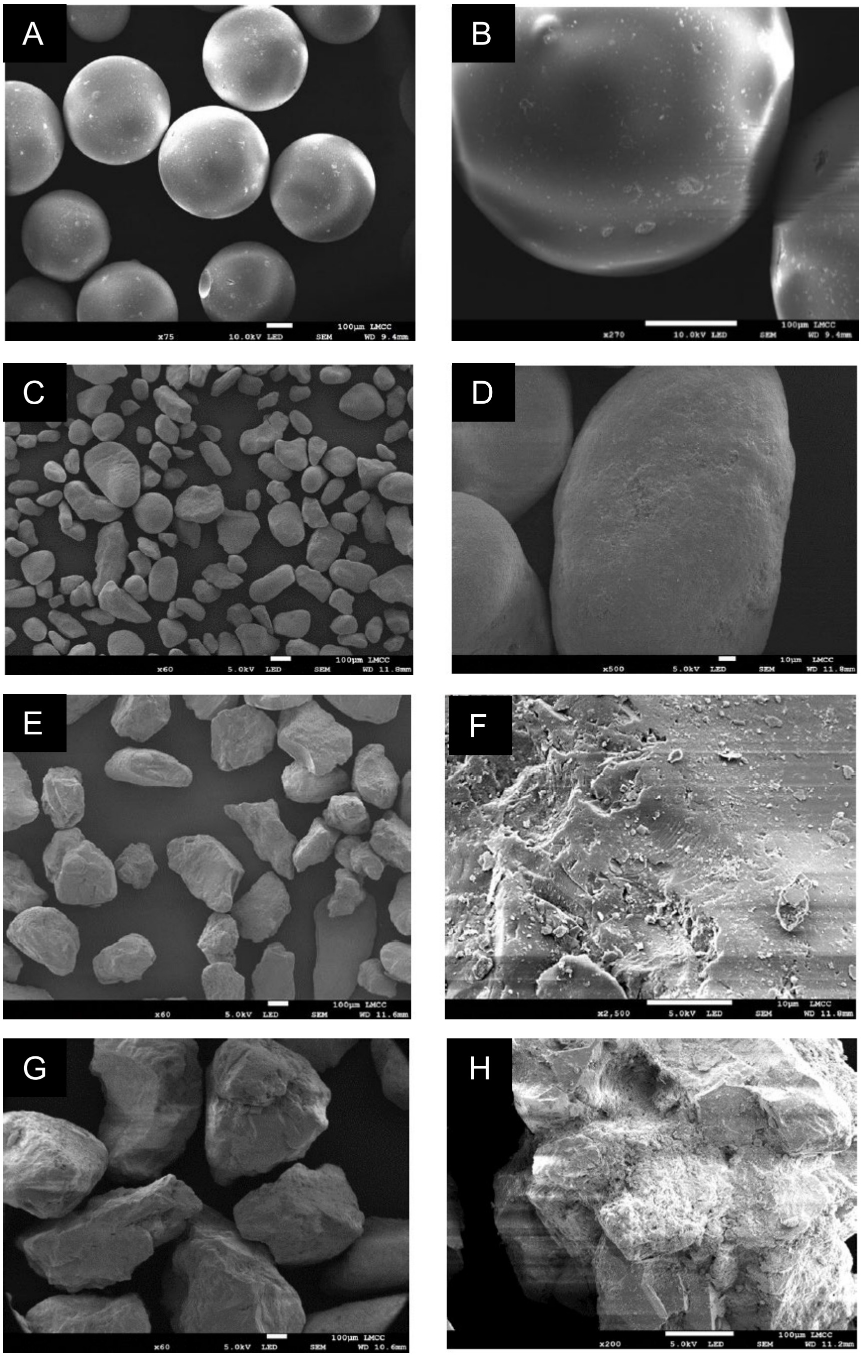

Supplementary Figure S2: Scanning electron micrographs at different magnifications of unabraded borexosilicate glass beads GB (A, B), desert sand SR_{149} (C,D), beach sand SA_{256} (E,F), commercial sand VA_{357} (G,H). For all images the white scale bar represents 100 μm.
